# Optimization of Pre-Analytical Handling to Maintain DNA Integrity in Diagnostic Papanicolaou Tests

**DOI:** 10.1016/j.jmoldx.2024.12.008

**Published:** 2025-01-17

**Authors:** Sara Schumacher, Jacob Malchau Lauesgaard, Therese Carlsson, Anna Linder, Karin Sundfeldt

**Affiliations:** ∗ Sahlgrenska Center for Cancer Research, Department of Obstetrics and Gynecology, Institute of Clinical Sciences, Sahlgrenska Academy at University of Gothenburg, Gothenburg, Sweden; † Sahlgrenska University Hospital, Region Västra Götaland, Gothenburg, Sweden; ‡ Sahlgrenska Center for Cancer Research, Department of Medical Chemistry and Cell Biology, Institute of Biomedicine, Sahlgrenska Academy at University of Gothenburg, Gothenburg, Sweden

## Abstract

Cell-free DNA (cfDNA) of ovarian carcinoma origin can be detected in samples from the gynecologic tract. This study aims to evaluate how pre-analytical handling affects DNA profile and integrity in Papanicolaou (Pap) tests, to optimize their potential for detection of ovarian cancers (OCs). Analysis of archived Pap tests from patients with OC, kept at room temperature for 48 hours and stored at −80°C, was complemented by *in vitro* experiments. Temperature-associated effects on DNA fragmentation were evaluated in samples stored at 4°C, −20°C, or −80°C. Time-dependent DNA degradation at room temperature was evaluated in comparison to storage at 4°C. Results were validated in prospectively collected Pap tests. The DNA integrity was assessed by fragment analysis. Accumulation of short DNA fragments was observed in archived Pap tests from patients with OC*. In vitro*, fragments of 100 to 350 bp increased 11.5-fold within 48 hours at room temperature compared with 1.7-fold when stored at 4°C. Consistent with the *in vitro* findings, prospectively collected samples showed reduced fragmentation when stored at 4°C compared with room temperature (*P* = 0.007). Long-term storage at 4°C had a significant negative effect on DNA stability (*P* = 0.013), whereas freezing slowed down fragmentation. Immediate storage at 4°C after sampling markedly reduces DNA degradation, suggesting a simple way to optimize pre-analytical handling and decrease unwanted fragmentation for cfDNA analysis in Pap tests.

There is an unmet need for earlier detection of various gynecologic conditions, among them, and most pressingly, ovarian cancers (OCs). Because of absent or subtle symptoms at early stages of disease, most patients with OC are diagnosed in advanced stages, leading to low curability and poor survival rates.

Early detection of cervical dysplasia and cancer, through endocervical cytologic sampling, known as the Papanicolaou (Pap) test, has significantly decreased the cervical cancer incidence in countries with organized screening.^1^ Efforts are made to use new molecular techniques (eg, next-generation sequencing) to enable early detection of other gynecologic malignancies in this and other gynecologic sample types. The original Pap test, as described by George Papanicolaou in 1941, involves smearing of collected cells onto glass slides, fixation, and staining, followed by examination under a microscope.^2^ Since the 1990s, classic smears have been replaced by liquid-based cytology using alcohol-based solutions for fixation.^3^^,^^4^ Currently, a further transition from cytologic diagnostics to detection of high-risk subtypes of human papillomavirus (HPV) is taking place within the field of screening for cervical cancer precursor lesions. With this, focus has again shifted from sampling in a solution meant to preserve cells for cytologic evaluation to solutions preserving DNA suitable for real-time quantitative PCR. However, because the Pap test is used as a confirmatory test and as the primary test of choice in many settings, its usability for molecular diagnostics remains a subject of interest.

Cell-free DNA (cfDNA) refers to fragments of DNA present in bodily fluids due to degradation of the cells of origin. cfDNA is continuously released through normal biological processes, such as apoptosis, necrosis, or active secretion.^5^ If OC is present, cells and cfDNA of tumor origin (ctDNA) harboring tumor-derived mutations may be transported through the fallopian tubes to the uterine cavity, the cervical canal, and the vagina. It is possible to detect ctDNA, using ultrasensitive methods and next generation sequencing, in Pap tests as well as endometrial samples, uterine lavages, and vaginal tampons.^6^, ^7^, ^8^ In plasma, one of the major challenges is the low fraction of ctDNA (≤0.1% of cfDNA), particularly in early-stage disease.^9^ Technical performance depends on DNA quality, as it has been demonstrated that post-sampling DNA fragmentation negatively affects ctDNA assay performance.^10^ Additionally, increasing evidence suggests that the unique and disease-specific fragmentation patterns of ctDNA may be valuable as a biomarker.^11^ It is, therefore, crucial to ensure that the DNA quality remains high, to avoid potential loss of valuable diagnostic information.

With the increasing number of specimens being introduced for cfDNA analysis, the effect of the sampling media and pre-analytical handling needs to be evaluated.^12^ The most common specimen used for cfDNA analysis is plasma, and pre-analytical handling of this sample type has previously been addressed.^13^^,^^14^ In comparison, Pap tests are poorly investigated in terms of pre-analytical considerations. Herein, we evaluate how the DNA profile and integrity are affected by handling and storage conditions before DNA extraction. Data are gathered from a series of *in vitro* samples, and from archived and prospectively collected Pap tests (Figure 1).

**Figure 1:**
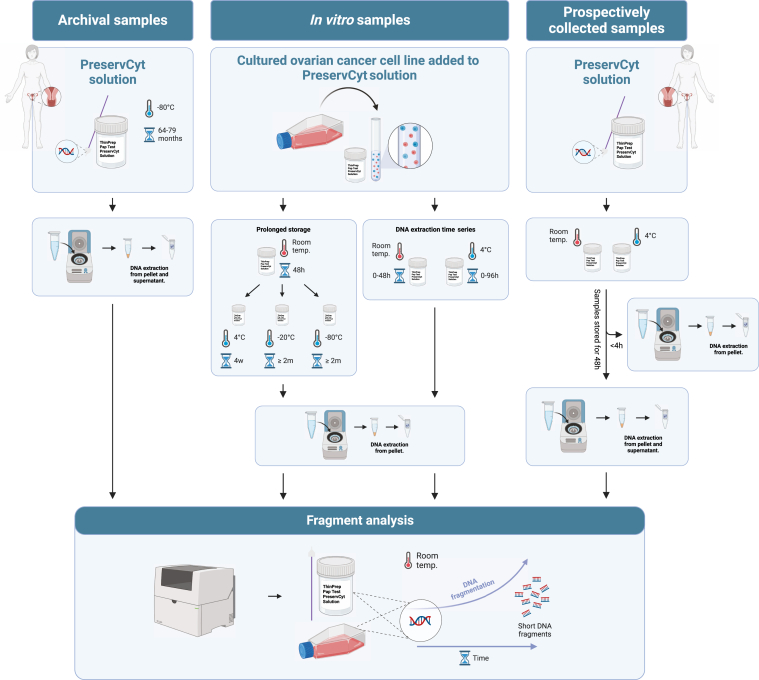
Schematic illustration of sample collection and experimental workflow. DNA was extracted from *in vitro* samples in addition to archived and prospectively collected Pap tests stored at different temperatures (room temperature, 4°C, −20°C, and −80°C). The DNA profiles were evaluated using fragment analysis. Created with BioRender.com (Toronto, ON, Canada).

## Materials and Methods

### Patients and Clinical Samples

Pap tests (*n* = 12) were collected from patients undergoing surgery for OC during 2016 at Sahlgrenska University Hospital, Gothenburg, Sweden (Supplemental Table S1). Sampling was done after the initiation of anesthesia but before vaginal cleansing. These samples were obtained with a cytobrush following routine procedure and kept in the methanol-based ThinPrep PreservCyt solution (catalog number PRD-07132; Hologic Inc., Marlborough, MA) at room temperature for 48 hours before being aliquoted and stored at −80°C for 64 to 79 months, without being subjected to freeze-thaw cycles. Henceforth, these samples are referred to as archived Pap tests.

Pap tests were also collected prospectively from patients enrolled at Sahlgrenska University Hospital from December 2022 to June 2023. Samples were collected in duplicates by trained gynecologists (J.M.L. and K.S.) from patients undergoing surgery for benign gynecologic conditions (*n* = 10) (Supplemental Table S2). Samples were collected in ThinPrep PreservCyt according to the same routine procedure used for the archived Pap tests. One sample was immediately placed at 4°C and one was kept at room temperature. Within 4 hours, the samples underwent centrifugation at 200 × *g* for 10 minutes. Pap tests, archived and prospectively collected, presented a pellet and a supernatant. The supernatant was temporarily removed, and the pellet was divided equally, one half for immediate DNA extraction (<4 hours after sampling) and one for DNA extraction after 48 hours. The supernatant was transferred back to the remaining cell pellet, and the sample was stored at room temperature or 4°C. After 48 hours, the samples were centrifuged at 200 × *g* for 10 minutes, and DNA from pellet and supernatant was extracted separately (Supplemental Table S3).

### Cell Culturing and *in Vitro* Sample Preparation

Human ovarian carcinoma cell line (SK-OV-3) was used in the *in vitro* experiments. Cells were cultured in Gibco McCoy's 5A (Modified) Medium (catalog number 16600082; Fisher Scientific, Pittsburgh, PA) supplemented with 10% fetal bovine serum (v/v; catalog number 10270106; ThermoFisher Scientific, Waltham, MA) and 1% penicillin/streptomycin (v/v; Invitrogen, Carlsbad, CA). Cells were washed with Gibco Dulbecco's phosphate-buffered saline (catalog number 14190144; ThermoFisher Scientific) and detached with Trypsin-EDTA (catalog number T4049; Sigma-Aldrich, St. Louis, MO). Cell viability of ≥80% was confirmed for all experiments. Clinical sampling was simulated by addition of ThinPrep PreservCyt solution to SK-OV-3 cells, followed by 48 hours of sedimentation at room temperature, simulating standard handling of Pap tests before analysis and biobanking. All samples had a final concentration of 200,000 cells/mL. DNA was extracted at 48 hours (control), or after further storage at 4°C (1 month), −20°C, or −80°C (≥2 months). For the DNA extraction time series, cells were left in ThinPrep solution for 0 to 48 hours (time = 0, 6, 12, 24, 36, and 48 hours) at room temperature or 0 to 96 hours (time = 0, 6, 12, 24, 36, 48, 60, 72, 84, and 96 hours) at 4°C. A lymphocyte cell line (K562) was used to validate the DNA fragmentation results (room temperature, time = 48 hours).

### DNA Extraction and Quantification

DNA was extracted according to the manufacturers' protocol with minor modifications. In brief, DNA was extracted from the pellet using the QIAmp DNA Micro Kit (catalog number 56304; Qiagen, Venlo, the Netherlands) and from the supernatant using the QIAmp Circulating Nucleic Acid Kit (catalog number 55114; Qiagen). DNA was quantified using the Qubit 1× dsDNA High Sensitivity Assay Kit (catalog number Q33230; Invitrogen) with a Qubit 3.0 Fluorometer. *In vitro* samples with a DNA concentration below the DNA integrity number (DIN) functional range were concentrated using an Eppendorf 5301 Vacufuge Vacuum Concentrator (Eppendorf, Hamburg, Germany) for 5 minutes at 45°C.

### Fragment Analysis

DNA integrity analysis was performed using 4200 TapeStation system (Agilent Technologies, Santa Clara, CA). Samples were prepared according to the manufacturers' protocols for High Sensitivity D5000 ScreenTape Assay and Genomic DNA ScreenTape Assay (catalog numbers 5067-5592 and 5067-5365; Agilent Technologies). Data were analyzed using Agilent TapeStation Software version 4.1.1 (Agilent Technologies). The amount of short DNA fragments was expressed as the percentage of the region of interest relative to the total DNA analyzed. Gating for region quantification was defined on the basis of the DNA fragment size profile, within the sizing range of the instrument, where the lower limit was 100 bp. DIN values were generated for paired patient samples with DNA concentrations above the lower limit of the DIN functional range (*n* = 20). DIN was generated for archived and prospectively collected samples when possible.

### Ethical Approval

Ethical approval was granted by the Regional Ethical Review Board in Gothenburg (number 510-13; amendment number T052-17; and number 2021-06368-01). Informed consent was obtained from all patients, and the project was conducted in line with the Declaration of Helsinki.

### Statistical Analysis

Statistical analyses were performed using GraphPad Prism version 9.0.0 for Windows (GraphPad Software, Boston, MA). All *in vitro* experiments were conducted in triplicate and presented as medians unless otherwise stated. Statistical testing of the long-term storage experiments was performed using the Kruskal-Wallis test, followed by Dunn's test for multiple comparisons. Differences in the time series extractions were evaluated using the Friedman test, followed by the Dunn's test or the *U-*test. Statistical testing of the clinical specimens was performed using the *U-*test, or for the comparison in DIN, the Wilcoxon signed-rank test. Data were considered statistically significant when ∗*P* < 0.05 and ∗∗*P* < 0.01.

## Results

### Presence of Short DNA Fragments in Archival Pap Tests

**Figure 2:**
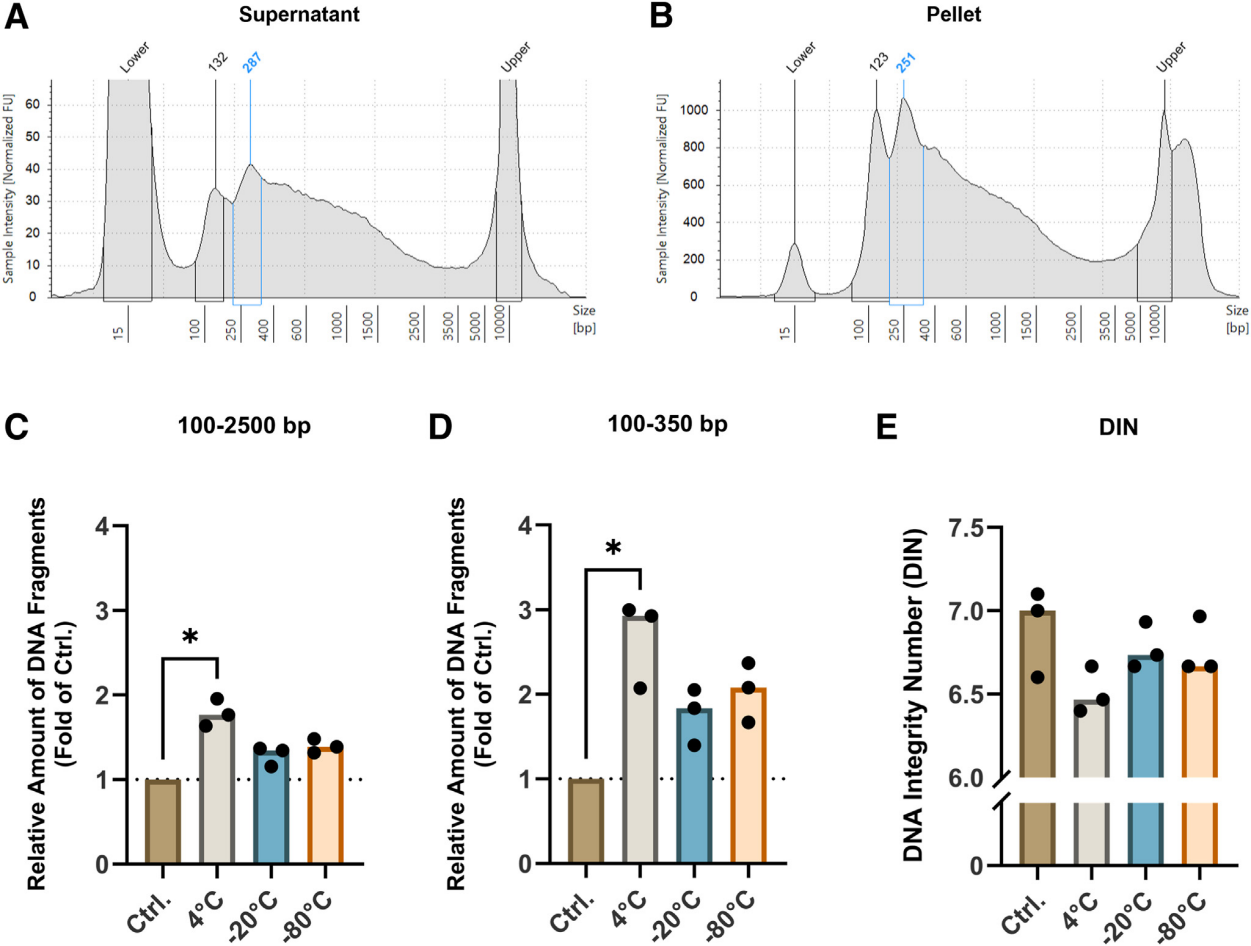
Temperature-dependent effects on DNA integrity. **A** and **B:** DNA profiles of the supernatant (**A**) and pellet (**B**) from an archival Pap test. DNA integrity analyses *in vitro* after 48 hours at room temperature [control (Ctrl.)], and after storage at 4°C, −20°C, or −80°C for ≥4 weeks. **Blue lines** and numbers indicate the main peak as identified by the TapeStation software version 4.1.1. **C** and **D:** Relative amount of DNA fragments within 100 to 2500 bp (**C**) and 100 to 350 bp (**D**). Controls were normalized to 1, indicated by **dotted lines**. **E:** DNA integrity number (DIN). ∗*P* < 0.05. FU, fluorescence units.

Initially, the archived Pap tests (*n* = 12) kept at room temperature for ≥48 hours before storage at −80°C for 5 to 6 years were analyzed. Accumulation of short DNA fragments (<2500 bp with distinct peaks at <300 bp) was observed in both the supernatant and the pellet in paired patient samples, and the two sample compartments exhibited similar DNA profiles (Figure 2, A and B). The short DNA fragments were visible in 12 of 12 pellets and in 5 of 11 supernatants. The median amount of DNA was 586.05 ng (range, 105.35 to 14,973.00 ng) in pellets and 33.08 ng (range, 13.62 to 117.01 ng) in supernatants.

### Long-Term Storage Temperature Affects the DNA Integrity

**Supplemental Figure S1:**
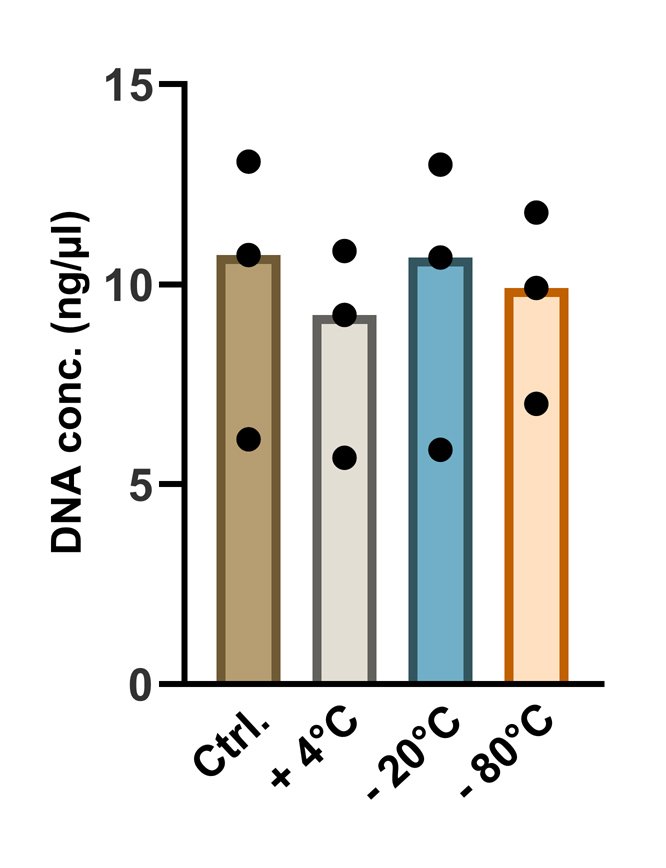
Median concentration (conc.) of extracted DNA from *in vitro* samples stored at various temperatures after initial storage at room temperature for 48 hours. Ctrl. denotes samples extracted directly after 48 hours. Dots denote range.

DNA fragmentation was evaluated *in vitro*, mimicking the clinical situation. Samples were aliquoted in methanol-based ThinPrep solution, kept at room temperature for 48 hours, and thereafter stored at different conditions, 4°C, −20°C, or −80°C for ≥4 weeks. Short DNA fragments were found in all samples, with a mean fragment size of 269 bp. DNA fragmentation was significantly increased within the regions of 100 to 2500 bp (*P* = 0.013) and 100 to 350 bp (*P* = 0.026) after storage at 4°C (Figure 2, C and D). No significant differences in DNA fragmentation were observed at −20°C or −80°C, although a tendency toward further degradation taking place at both −20°C and −80°C was observed. A decline in DIN was observed in 4°C samples, although this was not statistically significant (*P* = 0.26) (Figure 2E). DNA concentrations did not differ between storage temperatures (Supplemental Figure S1).

In conclusion, a significant accumulation of short DNA fragments was observed at 4°C, suggesting that long-term storage in −20°C or −80°C should be preferred. Moreover, all samples displayed similar DNA profiles, suggesting that DNA fragmentation commenced during the initial 48 hours when at room temperature.

### Temperature and Time-Dependent DNA Fragmentation *in Vitro*

**Figure 3:**
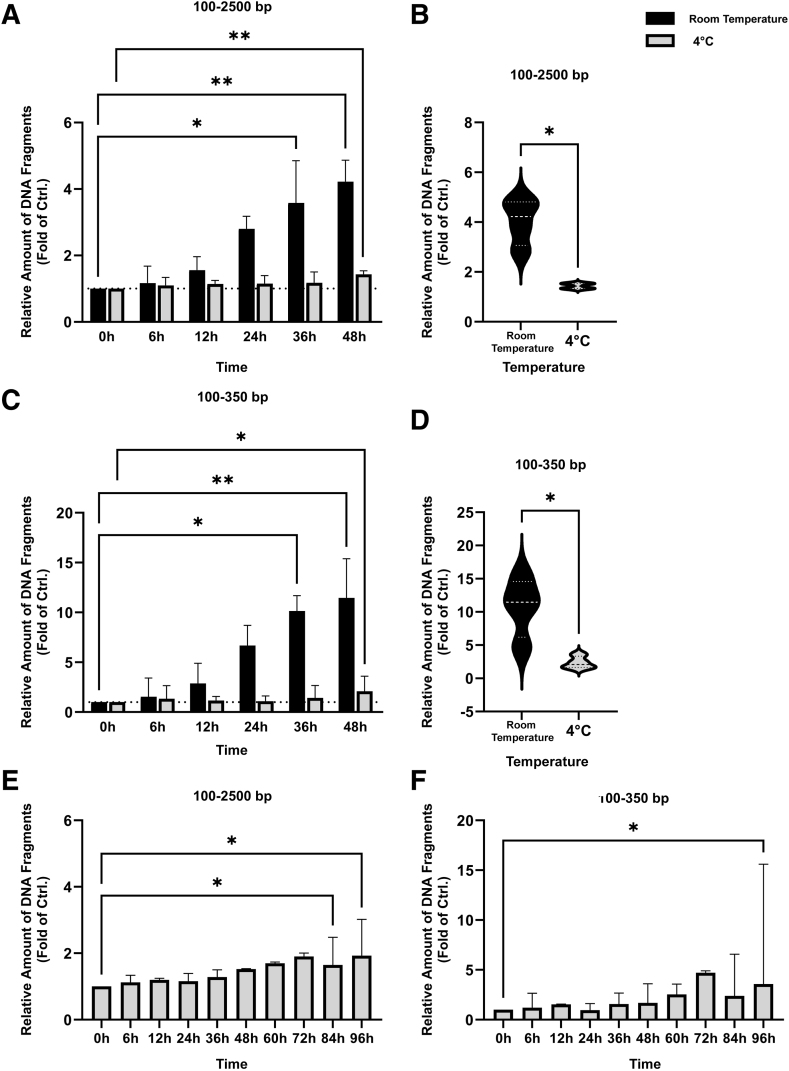
DNA fragmentation *in vitro*. Relative increase of DNA fragments (100 to 2500 bp) at room temperature (black) and 4°C (gray) for 0 to 48 hours (**A**), and between room temperature and 4°C at 48 hours (**B**). Relative increase (100 to 350 bp) at room temperature (black) and 4°C (gray) for 0 to 48 hours (**C**), and between room temperature and 4°C at 48 hours (**D**). Controls (Ctrls.) were normalized to 1, indicated by **dotted lines**. For the violin plots, the median and quartiles are indicated by **dotted lines**. DNA fragmentation within 100 to 2500 bp (**E**) and 100 to 350 bp (4°C, 0 to 96 hours; **F**). All data were normalized against a control (time = 0 hours). Data are given as means ± SD (**A**, **C**, **E**, and **F**). ∗*P* < 0.05, ∗∗*P* < 0.01.

**Supplemental Figure S2:**
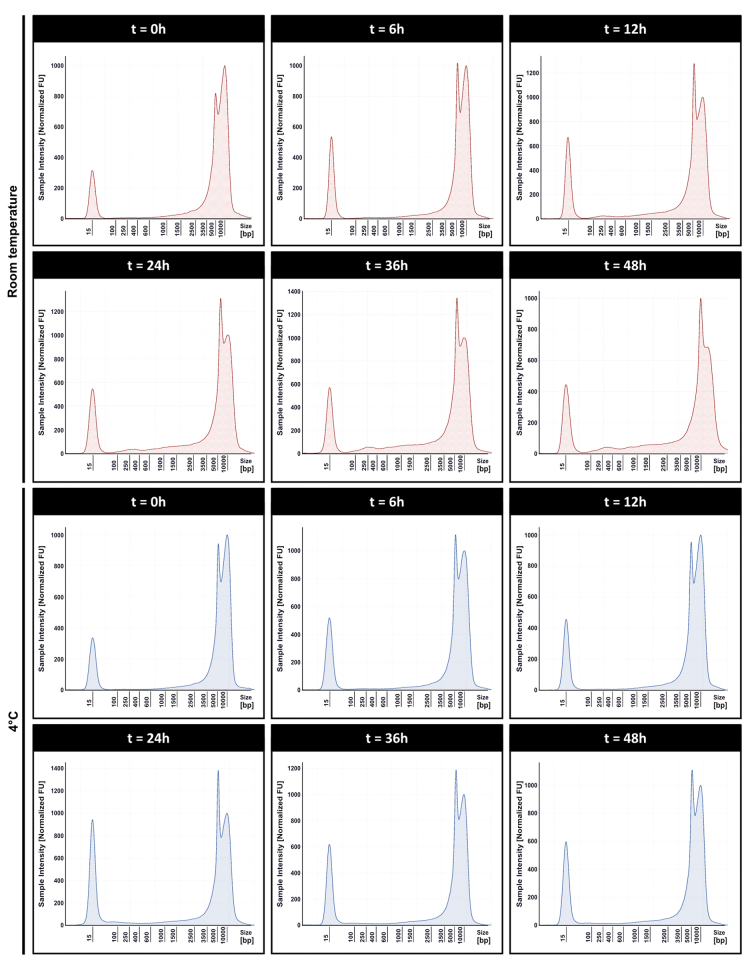
DNA fragmentation profiles of time series of *in vitro* samples [time (t) = 0 hours to t = 48 hours] at room temperature (**top**) and 4°C (**bottom**). FU, fluorescence units.

Next, time series (0 to 96 hours) were set up *in vitro* at room temperature and 4°C. The relative quantity of short DNA fragments gradually increased with time at room temperature (Figure 3A–D, and Supplemental Figure S2). The relative increase in short DNA fragments after 48 hours was significantly higher in samples stored at room temperature than 4°C within 100 to 2500 bp (*P* = 0.029) and 100 to 350 bp (*P* = 0.029) (Figure 3, B and D).

**Supplemental Figure S3:**
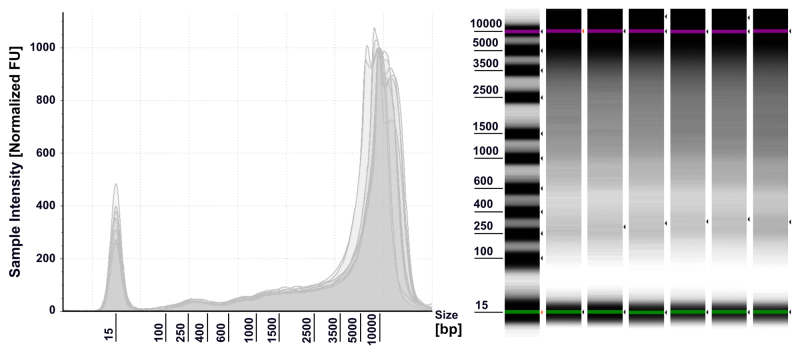
Electropherogram overlay (**left panel**) and gel image (**right panel**) of fragmentation analysis of K562 cell line validation samples (room temperature, time = 48 hours). Green and purple indicate the lower and upper markers, respectively. FU, fluorescence units.

At room temperature, time = 0 hours, gating for 100 to 2500 bp and 100 to 350 bp showed a median percentage of total of 6.16% and 0.39%, respectively (Figure 3, A and C). An accumulation of short DNA fragments was detectable within both gates already at 6 hours and statistically significant at 36 hours. After 48 hours, the amount of DNA fragments within these regions had increased to 24.19% and 4.75%, respectively. This was equivalent to a 4.2-fold increase for the 100- to 2500-bp region (*P* = 0.003) (Figure 3B), and an 11.5-fold increase for the 100- to 350-bp region (*P* = 0.002) (Figure 3D). A similar accumulation was observed for the K562 cell line (Supplemental Figure S3).

At 4°C, time = 0 hours, gating for 100 to 2500 bp and 100 to 350 bp showed a median percentage of total of 3.77% and 0.12%, respectively. After 48 hours, the amount of DNA fragments had increased to 5.74% and 0.34%, respectively. This was equivalent to a 1.5-fold increase for the 100- to 2500-bp region (*P* = 0.002) (Figure 3B), and a 1.7-fold increase for the 100- to 350-bp region (*P* = 0.03) (Figure 3D). After extending the time to 96 hours at 4°C, the median percentage of total increased to 7.28% (100 to 2500 bp) and 0.54% (100 to 350 bp), equivalent to a 1.9- and 3.6-fold increase, respectively (Figure 3, E and F).

In conclusion, accumulation of short DNA fragments *in vitro* was significantly haltered if samples were immediately placed at 4°C. Furthermore, this strongly suggests that the accumulation of short DNA fragments observed in the archived Pap tests was initiated before freeze storage.

### Temperature-Dependent DNA Fragmentation in Prospectively Collected Pap Tests

**Figure 4:**
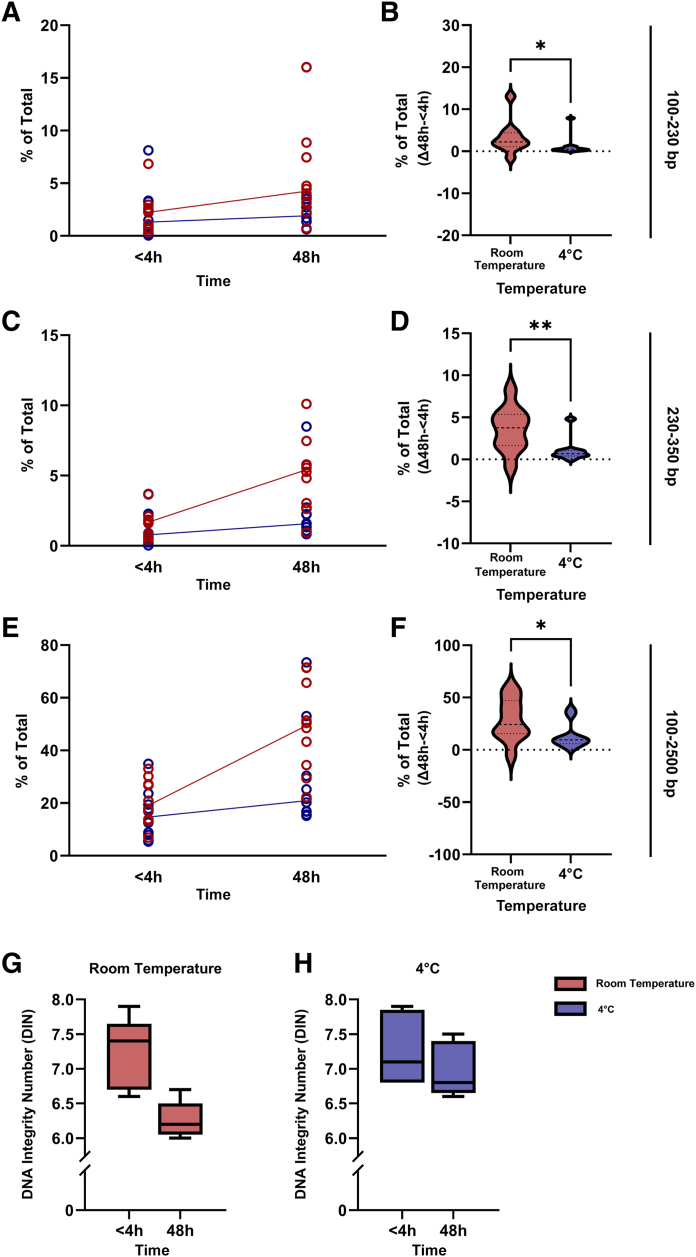
DNA fragmentation in prospectively collected Pap tests. DNA extracted from the pellet fraction of Pap tests stored at room temperature (red) or 4°C (blue) for <4 or 48 hours, analyzed with TapeStation. **A**–**F:** DNA fragment quantification within 100 to 230 bp (**A** and **B**) for <4 and 48 hours (**A**) and 48 hours (**B**), 230 to 350 bp (**C** and **D**) for <4 and 48 hours (**C**) and 48 hours (**D**), and 100 to 2500 bp (**E** and **F**) for <4 and 48 hours (**E**) and 48 hours (**F**). **Lines** connect the medians. For the violin plots, the median and quartiles are indicated by **dotted lines**. **G** and **H:** DNA integrity number (DIN). Box plots show minimum and maximum values. *n* = 10 patients (**A**–**F**); *n* = 5 (**G** and **H**). ∗*P* < 0.05, ∗∗*P* < 0.01.

Next, the *in vitro* findings were tested on prospectively collected patient Pap tests. The samples were kept at room temperature or 4°C, and the cell pellet fraction was analyzed within 4 hours and at 48 hours from sample collection. The total DNA yield from the pellets was higher compared with the corresponding supernatants (median, 1.61 versus 0.06 μg) (Supplemental Table S3). The relative increase in short DNA fragments was significantly higher in samples stored at room temperature than 4°C within 100 to 2500 bp (*P* = 0.03), 100 to 230 bp (*P* = 0.02), and 230 to 350 bp (*P* = 0.007) (Figure 4, A–F). The increase of short DNA fragments at room temperature was larger within the region of 230 to 350 bp than that of 100 to 230 bp, suggesting a slight shift in peaks toward higher molecular weight fragments. Consistent with the *in vitro* samples, clinical samples stored at room temperature for 48 hours demonstrated a higher level of fragmentation within the regions of 100 to 350 bp (*P* = 0.02) and 100 to 2500 bp (*P* = 0.02) compared with samples stored at 4°C. Samples stored at room temperature displayed a median DIN of 7.4 at <4 hours and 6.2 at 48 hours, whereas samples stored at 4°C had a median DIN of 7.1 at <4 hours and 6.8 at 48 hours (Figure 4, G and H). The negative change in median DIN was four times larger at room temperature compared with samples stored at 4°C; however, this difference did not reach statistical significance (*P* = 0.06).

In conclusion, in prospectively collected Pap tests, DNA fragmentation was significantly haltered in samples immediately placed at 4°C, confirming the results obtained from the *in vitro* analyses. Moreover, the DNA fragments were slightly larger in samples stored at room temperature, suggesting further fragmentation of shorter fragments along with accumulation of larger fragments.

## Discussion

Liquid biopsies are minimally invasive, easily accessible, and provide a more comprehensive molecular profile of a tumor than a single tissue biopsy.^15^ Sampling in close anatomic proximity to the tumor of origin should increase the yield of ctDNA. This is supported by the high occurrence of tumor-derived mutations in ovarian cyst fluid.^16^ Moreover, the anatomic proximity when sampling from the endocervix should also increase probability that observed ctDNA originates from the reproductive organs.

Short DNA fragments are of particular importance in cfDNA detection techniques, and evidence is building that the size distribution profile of cfDNA can be used to discriminate between healthy patients and patients with cancer.^11^ The tumor-derived fraction of cfDNA (ctDNA) is often small, especially at early stages of disease. Therefore, it is essential that the integrity of sampled DNA is maintained, and that unwanted fragmentation and cellular contamination are avoided.

Numerous studies have evaluated the feasibility of detecting ctDNA from OC in noninvasive samples from the gynecologic tract, mostly Pap tests collected in ThinPrep solution.^6^^,^^17^, ^18^, ^19^, ^20^, ^21^, ^22^ The methods of analysis differ between the studies, but the usability in a potential screening setting has generally been low. This has mainly been because of unsatisfying sensitivity and/or specificity, tumor mutational profile dependency, or a combination of both. The prevalence of OC is low, which increases the demand for optimal specificity in a screening setting involving large populations. Suboptimal positive predictive values will lead to patient anxiety and unnecessary invasive follow-up. None of the aforementioned studies address the potential suboptimal pre-analytical handling of samples, although Paracchini et al^19^ (2023) suggest that their observed low sensitivity could be because of sampling and storage conditions. Krimmel-Morrison et al^21^ stated that Pap testing was performed according to the manufacturer's instructions, and samples were thereafter frozen at −80°C, similarly to the archived samples in this article. One should keep in mind that the manufacturer's instructions when it comes to ThinPrep are optimized for cytologic preservation and not preservation of cfDNA.

Evaluation of DNA isolated from archived Pap tests stored at −80°C indicated an accumulation of short DNA fragments, not fully corresponding to, but overlapping with cfDNA fragments with the apoptosis-associated average size of approximately 167 bp (a mononucleosome and an approximately 20-bp linker).^23^^,^^24^ Accumulation of short DNA fragments was noted in both the supernatant and the pellet of the samples, potentially obscuring and preventing assessment of cfDNA in the sample based on fragment size. We hypothesized that the DNA fragmentation pattern of the archived Pap tests could be explained by pre-analytical factors rather than reflecting the DNA profiles at time of collection. Confirming this, a gradual increase of short DNA fragments *in vitro* was observed within the initial 48 hours in methanol-based preservative solution at room temperature. A tendency toward continuous degradation of DNA in *in vitro* samples stored in the freezer was also observed. This tendency could contribute to the suboptimal results in the study by Krimmel-Morrison et al^21^ and others analyzing samples stored at subzero temperatures.

Inconclusive results have previously been reported regarding DNA stability in Pap tests. It is commonly accepted that Pap tests can be the subject of PCR-based HPV analysis, and Kim et al^25^ were able to perform multiplex PCR on Pap tests stored 1 year at room temperature. Lin et al^3^ were able to detect HPV DNA in Pap tests stored at ambient temperatures for up to 2 years. Chang et al^26^ were able to detect mutated DNA in the cell pellet of fine-needle aspirations stored in ThinPrep for up to 1 year. Meanwhile, Castle et al^27^ showed that long-term storage of cervical samples at ambient temperatures decreased HPV DNA stability, and that HPV DNA quality correlated negatively with storage time. None of the studies reported integrity and fragmentation profile of cfDNA. The data presented in this article showed that the DNA integrity *in vitro* and in prospectively collected Pap tests decreased significantly within the first few hours after sampling and continuously declined over time. Importantly, the data also showed that before extraction, DNA degradation could be haltered by decreasing the storage temperature to 4°C immediately. The maintenance of DNA integrity is of utmost importance for detection of rare DNA species, such as ctDNA.^10^ Thus, the results encourage immediate cooling of Pap tests to ensure high DNA quality when cfDNA is the target molecule of analysis, because enrichment of molecules at this specific size, 100 to 230 bp, was significant.

One prominent strength of the study was the short time between sample collection at the clinic and DNA analysis, as that minimizes factors potentially influencing the DNA profile of the sample. To our knowledge, this is the only study on pre-analytical sample handling and DNA integrity from a cfDNA perspective. A limitation was the need for separate collection tubes for room temperature and 4°C clinical samples as this prevented direct comparison of DNA fragmentation over time. However, immediate subdivision of Pap tests into different storage conditions was not feasible in the clinical setting. Another limitation might be the small number of samples. However, the decision to include 10 patients was made in advance as this number was deemed sufficient should the changes in sample handling be enough to significantly improve DNA quality.

## Conclusion

Analysis of tumor-derived cfDNA in Pap tests could hold the potential to revolutionize screening for early stages of OC. Several studies have sought to establish a credible method without reaching sufficient sensitivity and specificity. This study highlights the importance of optimized procedures for sampling and pre-analytical handling of specimens from the gynecologic tract, when working to expand the spectrum of diagnostic use of such samples. The findings strongly suggest that the DNA integrity of Pap tests exhibits superior preservation if kept at 4°C instead of room temperature immediately after sampling. Furthermore, the data indicate that DNA of Pap tests is relatively but not completely stable if the sample is frozen. The common clinical practice of allowing Pap tests to be stored at room temperature for up to 48 hours after sampling seems to affect DNA integrity negatively, specifically obscuring the fragmentation within the size range of 100 to 230 bp that is of particular interest in ctDNA analysis.

## Disclosure Statement

None declared.

## References

[1] Peirson L., Fitzpatrick-Lewis D., Ciliska D., Warren R. (2013). Screening for cervical cancer: a systematic review and meta-analysis. Syst Rev.

[2] Papanicolaou G.N., Traut H.F. (1941). The diagnostic value of vaginal smears in carcinoma of the uterus. Am J Obstet Gynecol.

[3] Lin W.M., Ashfaq R., Michalopulos E.A., Maitra A., Gazdar A.F., Muller C.Y. (2000). Molecular Papanicolaou tests in the twenty-first century: molecular analyses with fluid-based Papanicolaou technology. Am J Obstet Gynecol.

[4] Sherman M.E., Schiffman M.H., Lorincz A.T., Herrero R., Hutchinson M.L., Bratti C., Zahniser D., Morales J., Hildesheim A., Helgesen K., Kelly D., Alfaro M., Mena F., Balmaceda I., Mango L., Greenberg M. (1997). Cervical specimens collected in liquid buffer are suitable for both cytologic screening and ancillary human papillomavirus testing. Cancer Cytopathol.

[5] Thierry A.R., El Messaoudi S., Gahan P.B., Anker P., Stroun M. (2016). Origins, structures, and functions of circulating DNA in oncology. Cancer Metastasis Rev.

[6] Kinde I., Bettegowda C., Wang Y., Wu J., Agrawal N., Shih Ie M., Kurman R., Dao F., Levine D.A., Giuntoli R., Roden R., Eshleman J.R., Carvalho J.P., Marie S.K., Papadopoulos N., Kinzler K.W., Vogelstein B., Diaz L.A. (2013). Evaluation of DNA from the Papanicolaou test to detect ovarian and endometrial cancers. Sci Transl Med.

[7] Maritschnegg E., Wang Y., Pecha N., Horvat R., Van Nieuwenhuysen E., Vergote I., Heitz F., Sehouli J., Kinde I., Diaz L.A., Papadopoulos N., Kinzler K.W., Vogelstein B., Speiser P., Zeillinger R. (2015). Lavage of the uterine cavity for molecular detection of müllerian duct carcinomas: a proof-of-concept study. J Clin Oncol.

[8] Erickson B.K., Kinde I., Dobbin Z.C., Wang Y., Martin J.Y., Alvarez R.D., Conner M.G., Huh W.K., Roden R.B.S., Kinzler K.W., Papadopoulos N., Vogelstein B., Diaz L.A., Landen C.N. (2014). Detection of somatic TP53 mutations in tampons of patients with high-grade serous ovarian cancer. Obstet Gynecol.

[9] Bettegowda C., Sausen M., Leary R.J., Kinde I., Wang Y., Agrawal N. (2014). Detection of circulating tumor DNA in early- and late-stage human malignancies. Sci Transl Med.

[10] Johansson G., Andersson D., Filges S., Li J., Muth A., Godfrey T.E., Ståhlberg A. (2019). Considerations and quality controls when analyzing cell-free tumor DNA. Biomol Detect Quantif.

[11] Cristiano S., Leal A., Phallen J., Fiksel J., Adleff V., Bruhm D.C. (2019). Genome-wide cell-free DNA fragmentation in patients with cancer. Nature.

[12] Tivey A., Church M., Rothwell D., Dive C., Cook N. (2022). Circulating tumour DNA — looking beyond the blood. Nat Rev Clin Oncol.

[13] Markus H., Contente-Cuomo T., Farooq M., Liang W.S., Borad M.J., Sivakumar S., Gollins S., Tran N.L., Dhruv H.D., Berens M.E., Bryce A., Sekulic A., Ribas A., Trent J.M., LoRusso P.M., Murtaza M. (2018). Evaluation of pre-analytical factors affecting plasma DNA analysis. Sci Rep.

[14] van der Pol Y., Moldovan N., Verkuijlen S., Ramaker J., Boers D., Onstenk W., de Rooij J., Bahce I., Pegtel D.M., Mouliere F. (2022). The effect of preanalytical and physiological variables on cell-free DNA fragmentation. Clin Chem.

[15] Siravegna G., Marsoni S., Siena S., Bardelli A. (2017). Integrating liquid biopsies into the management of cancer. Nat Rev Clin Oncol.

[16] Wang Y., Sundfeldt K., Mateoiu C., Shih Ie M., Kurman R.J., Schaefer J., Silliman N., Kinde I., Springer S., Foote M., Kristjansdottir B., James N., Kinzler K.W., Papadopoulos N., Diaz L.A., Vogelstein B. (2016). Diagnostic potential of tumor DNA from ovarian cyst fluid. Elife.

[17] Wang Y., Li L., Douville C., Cohen J.D., Yen T.T., Kinde I. (2018). Evaluation of liquid from the Papanicolaou test and other liquid biopsies for the detection of endometrial and ovarian cancers. Sci Transl Med.

[18] Arildsen N.S., Martin de la Fuente L., Måsbäck A., Malander S., Forslund O., Kannisto P., Hedenfalk I. (2019). Detecting TP53 mutations in diagnostic and archival liquid-based Pap samples from ovarian cancer patients using an ultra-sensitive ddPCR method. Sci Rep.

[19] Paracchini L., Mannarino L., Romualdi C., Zadro R., Beltrame L., Fuso Nerini I., Zola P., Laudani M.E., Pagano E., Giordano L., Fruscio R., Landoni F., Franceschi S., Dalessandro M.L., Canzonieri V., Bocciolone L., Lorusso D., Bosetti C., Raspagliesi F., Garassino I.M.G., D'Incalci M., Marchini S. (2023). Genomic instability analysis in DNA from Papanicolaou test provides proof-of-principle early diagnosis of high-grade serous ovarian cancer. Sci Transl Med.

[20] Ghezelayagh T.S., Kohrn B.F., Fredrickson J., Manhardt E., Radke M.R., Katz R., Gray H.J., Urban R.R., Pennington K.P., Liao J.B., Doll K.M., Simons E.J., Burzawa J.K., Goff B.A., Speiser P., Swisher E.M., Norquist B.M., Risques R.A. (2022). Uterine lavage identifies cancer mutations and increased TP53 somatic mutation burden in individuals with ovarian cancer. Cancer Res Commun.

[21] Krimmel-Morrison J.D., Ghezelayagh T.S., Lian S., Zhang Y., Fredrickson J., Nachmanson D., Baker K.T., Radke M.R., Hun E., Norquist B.M., Emond M.J., Swisher E.M., Risques R.A. (2020). Characterization of TP53 mutations in Pap test DNA of women with and without serous ovarian carcinoma. Gynecol Oncol.

[22] Paracchini L., Pesenti C., Delle Marchette M., Beltrame L., Bianchi T., Grassi T., Buda A., Landoni F., Ceppi L., Bosetti C., Paderno M., Adorni M., Vicini D., Perego P., Leone B.E., D'Incalci M., Marchini S., Fruscio R. (2020). Detection of TP53 clonal variants in Papanicolaou test samples collected up to 6 years prior to high-grade serous epithelial ovarian cancer diagnosis. JAMA Netw Open.

[23] Lo Y.M., Chan K.C., Sun H., Chen E.Z., Jiang P., Lun F.M., Zheng Y.W., Leung T.Y., Lau T.K., Cantor C.R., Chiu R.W. (2010). Maternal plasma DNA sequencing reveals the genome-wide genetic and mutational profile of the fetus. Sci Transl Med.

[24] Snyder M.W., Kircher M., Hill A.J., Daza R.M., Shendure J. (2016). Cell-free DNA comprises an in vivo nucleosome footprint that informs its tissues-of-origin. Cell.

[25] Kim Y., Choi K.R., Chae M.J., Shin B.K., Kim H.K., Kim A., Kim B-h (2013). Stability of DNA, RNA, cytomorphology, and immunoantigenicity in residual ThinPrep® specimens. APMIS.

[26] Chang H., Lee H., Yoon S.O., Kim H., Kim A., Kim B-h (2012). BRAFV600E mutation analysis of liquid-based preparation–processed fine needle aspiration sample improves the diagnostic rate of papillary thyroid carcinoma. Hum Pathol.

[27] Castle P.E., Solomon D., Hildesheim A., Herrero R., Concepcion Bratti M., Sherman M.E., Cecilia Rodriguez A., Alfaro M., Hutchinson M.L., Terence Dunn S., Kuypers J., Schiffman M. (2003). Stability of archived liquid-based cervical cytologic specimens. Cancer.

