# Supplementary material for: Optimization of Pre-Analytical Handling to Maintain DNA Integrity in Diagnostic Papanicolaou Tests
Source: J Mol Diagn. 2025 Jan 17;27(3):199–208. doi: 10.1016/j.jmoldx.2024.12.008 (PMC12179505; doi:10.1016/j.jmoldx.2024.12.008)
Supplement: Supplemental Table S1 [file mmc1.docx]

**Supplemental Table S1**. Clinical archival Pap tests collected from patients with ovarian cancer.

| **Patient** | **Primary location** | **Histology** | **PAD** | **FIGO stage** |
| --- | --- | --- | --- | --- |
| **Papanicolaou test (ThinPrep PreservCyt)** | | | | |
| Patient 1 | Ovary | Serous | High-grade serous carcinoma | 3C |
| Patient 2 | Ovary | Serous | High-grade serous carcinoma | 3C |
| Patient 3 | Peritoneal | Serous | High-grade serous carcinoma - peritoneum (omentum) | 3C |
| Patient 4 | Ovary | Serous | High-grade serous carcinoma | 3C |
| Patient 5 | Fallopian tube | Serous | High-grade serous carcinoma | 3C |
| Patient 6 | Ovary | Serous | High-grade serous carcinoma | 3C |
| Patient 7 | Ovary | Serous | High-grade serous carcinoma | 4A |
| Patient 8 | Ovary | Serous | High-grade serous carcinoma | 4B |
| Patient 9 | Ovary | Serous | High-grade serous carcinoma | 3C |
| Patient 10 | Ovary | Endometrioid | Endometrioid carcinoma | 3C |
| Patient 11 | Fallopian tube | Serous | High-grade serous carcinoma | 3C |
| Patient 12 | Ovary | Serous | High-grade serous carcinoma | 2B |
