# Supplementary material for: Optimization of Pre-Analytical Handling to Maintain DNA Integrity in Diagnostic Papanicolaou Tests
Source: J Mol Diagn. 2025 Jan 17;27(3):199–208. doi: 10.1016/j.jmoldx.2024.12.008 (PMC12179505; doi:10.1016/j.jmoldx.2024.12.008)
Supplement: Supplemental Table S2 [file mmc2.docx]

**Supplemental Table S2.** Prospectively collected clinical Pap tests.

| **Patient** | **Age** | **Diagnosis** | **Location** | **#ENZIAN score** | |
| --- | --- | --- | --- | --- | --- |
| **Papanicolaou test (ThinPrep PreservCyt)** | | | | |  |
| Patient 1 | 34 | Endometriosis | Light adherences, no deep endometriosis | P1, O1/0, T1/0 | |
| Patient 2 | 45 | Endometriosis | Cesaerean scar endometriosis | N/A | |
| Patient 3 | 31 | Endometriosis | Endometrioma >7cm, Rectovaginal deep endometriosis, Adenomyosis | P2, O1/3, A3, B2/0, C1, FA | |
| Patient 4 | 39 | Pelvic pain, No endometriosis | N/A | N/A | |
| Patient 5 | 42 | Endometriosis, Leiomyoma | Deep rectal endometriosis, Adenomyosis, Leiomyoma | P3, B3/3, C3, FA | |
| Patient 6 | 44 | Endometriosis | Mild endometriosis of the fallopian tubes, Mild peritoneal endometriosis | P1, T1/1 | |
| Patient 7 | 28 | Endometriosis | Endometrioma 7cm, Peritoneal endometriosis, Adherences | P2, O2/1, T1/1 | |
| Patient 8 | 30 | Endometriosis | Endometrioma/hematosalpinx 6cm, Infection/inflammation, Frozen pelvis, Adenomyosis | P3, O2/0, T3/3, A2, B2/2, C2, FA | |
| Patient 9 | 38 | Endometriosis | Cesaerean scar endometriosis | N/A | |
| Patient 10 | 30 | Endometriosis | Endometrioma 8cm, Peritoneal endometriosis | P3, O3/1, B0/1 | |
