# Supplementary material for: Optimization of Pre-Analytical Handling to Maintain DNA Integrity in Diagnostic Papanicolaou Tests
Source: J Mol Diagn. 2025 Jan 17;27(3):199–208. doi: 10.1016/j.jmoldx.2024.12.008 (PMC12179505; doi:10.1016/j.jmoldx.2024.12.008)
Supplement: Supplemental Table S3 [file mmc3.docx]

**Supplemental Table S3.** Clinical prospectively collected Pap tests from patients undergoing surgery for benign conditions. Data was generated by fragmentation analysis using TapeStation.

|  |  | **Pellet (<4h)** | | | | | **Pellet (48h)** | | | | | **Supernatant (48h)** | | | | |
| --- | --- | --- | --- | --- | --- | --- | --- | --- | --- | --- | --- | --- | --- | --- | --- | --- |
| **Patient** | **Sample** | **DNA yield (µg)** | **DNA input TapeStation (ng)** | **% of total (100-230bp)** | **% of total (230-350bp)** | **% of total (100-2500bp)** | **DNA yield (µg)** | **DNA input TapeStation (ng)** | **% of total (100-230bp)** | **% of total (230-350bp)** | **% of total (100-2500bp)** | **DNA yield (µg)** | **DNA input TapeStation (ng)** | **% of total (100-230bp)** | **% of total (230-350bp)** | **% of total (100-2500bp)** |
| **Endocervical samples (ThinPrep PreservCyt)** | | | | | | | | | | | | | | | | |
| Patient 1 | RT | 1.83 | 6.14 | 0.33 | 0.63 | 13.85 | 5.60 | 4.74 | 4.46 | 5.79 | 51.39 | 1.25 | 16.60 | 5.86 | 7.76 | 72.24 |
|  | 4°C | 9.54 | 6.20 | 0.19 | 0.23 | 7.87 | 5.20 | 6.50 | 0.72 | 1.08 | 16.79 | 0.12 | 1.57 | 4.29 | 2.76 | 42.78 |
| Patient 2 | RT | 10.29 | 8.54 | 2.62 | 2.11 | 30.15 | 1.61 | 6.00 | 4.75 | 5.56 | 48.48 | 0.12 | 1.64 | 6.21 | 4.73 | 55.56 |
|  | 4°C | 10.71 | 7.28 | 3.34 | 2.28 | 23.63 | 1.78 | 5.92 | 3.51 | 2.26 | 25.24 | 0.22 | 2.96 | 9.97 | 6.95 | 71.34 |
| Patient 3 | RT | 1.87 | 5.36 | 0.75 | 0.75 | 13.17 | 1.60 | 6.14 | 3.09 | 4.82 | 43.27 | 0.04 | 0.50 | 9.51 | 6.05 | 44.03 |
|  | 4°C | 3.24 | 6.06 | 0.04 | 0.03 | 5.85 | 2.04 | 7.30 | 0.62 | 0.83 | 16.93 | 0.17 | 2.28 | 2.27 | 2.21 | 52.35 |
| Patient 4 | RT | 0.71 | 5.50 | 1.10 | 0.86 | 13.29 | 0.40 | 6.00 | 2.28 | 2.63 | 29.45 | 0.04 | 0.60 | 10.89 | 7.14 | 48.43 |
|  | 4°C | 1.58 | 5.08 | 1.11 | 0.66 | 8.86 | 0.36 | 6.14 | 1.38 | 0.99 | 15.18 | 0.04 | 0.55 | 10.27 | 6.84 | 49.11 |
| Patient 5 | RT | 1.95 | 6.38 | 2.27 | 1.61 | 17.27 | 1.02 | 6.58 | 7.45 | 7.47 | 71.53 | 0.01 | 0.13 | 7.50 | 3.88 | 15.93 |
|  | 4°C | 1.02 | 6.32 | 2.40 | 2.17 | 19.4 | 1.57 | 5.48 | 2.70 | 2.73 | 53.01 | 0.01 | 0.15 | 5.04 | 1.77 | 9.95 |
| Patient 6 | RT | 0.34 | 6.38 | 2.41 | 1.74 | 20.88 | 0.23 | 4.64 | 3.24 | 3.05 | 34.46 | 0.01 | 0.15 | 5.91 | 2.31 | 11.38 |
|  | 4°C | 0.14 | 2.84 | 0.97 | 0.44 | 5.34 | 0.29 | 5.84 | 2.14 | 1.52 | 15.65 | 0.01 | 0.15 | 6.12 | 2.84 | 12.40 |
| Patient 7 | RT | 1.31 | 6.10 | 0.62 | 0.43 | 6.92 | 2.58 | 5.88 | 4.03 | 5.27 | 65.67 | 0.05 | 0.65 | 3.69 | 3.79 | 45.86 |
|  | 4°C | 0.62 | 5.86 | 3.26 | 1.73 | 12.54 | 1.71 | 6.22 | 3.78 | 2.23 | 20.21 | 0.10 | 1.31 | 14.70 | 7.93 | 56.45 |
| Patient 8 | RT | 0.49 | 6.84 | 6.84 | 3.66 | 33.03 | 0.29 | 5.80 | 8.86 | 5.74 | 50.42 | 0.01 | 0.19 | 10.50 | 6.94 | 55.18 |
|  | 4°C | 1.03 | 6.62 | 8.12 | 3.69 | 34.89 | 0.39 | 7.70 | 16.02 | 8.49 | 73.44 | 0.02 | 0.29 | 10.75 | 6.55 | 66.39 |
| Patient 9 | RT | 1.12 | 6.44 | 2.22 | 1.83 | 27.16 | 3.30 | 6.60 | 0.70 | 0.95 | 22.26 | 0.14 | 1.90 | 4.10 | 3.98 | 60.65 |
|  | 4°C | 2.94 | 5.80 | 0.38 | 0.50 | 17.83 | 4.77 | 7.76 | 1.36 | 1.61 | 30.38 | 0.15 | 1.97 | 3.41 | 3.49 | 62.09 |
| Patient 10 | RT | 1.47 | 4.64 | 2.95 | 1.86 | 26.74 | 0.65 | 5.92 | 16.00 | 10.11 | 71.39 | 0.07 | 0.89 | 6.06 | 3.38 | 53.02 |
|  | 4°C | 2.48 | 6.20 | 1.49 | 0.91 | 16.81 | 2.30 | 6.64 | 1.69 | 1.33 | 21.56 | 0.22 | 2.96 | 6.33 | 4.09 | 61.82 |
| Mean | RT | 2.14 | 6.23 | 2.21 | 1.55 | 20.25 | 1.73 | 5.98 | 5.49 | 5.14 | 48.83 | 0.17 | 2.32 | 7.02 | 5.00 | 46.23 |
|  | 4°C | 3.33 | 6.16 | 2.13 | 1.26 | 15.30 | 2.04 | 6.50 | 3.39 | 2.31 | 28.84 | 0.11 | 1.42 | 7.31 | 4.54 | 48.47 |
| Median | RT | 1.39 | 6.26 | 2.25 | 1.68 | 19.08 | 1.31 | 6.00 | 4.25 | 5.42 | 49.45 | 0.05 | 0.62 | 6.14 | 4.36 | 50.73 |
|  | 4°C | 2.03 | 6.20 | 1.30 | 0.79 | 14.68 | 1.75 | 6.36 | 1.92 | 1.57 | 20.89 | 0.11 | 1.44 | 6.23 | 3.79 | 54.40 |
